# Cree Food Knowledge and Being Well

**DOI:** 10.3390/ijerph22020181

**Published:** 2025-01-28

**Authors:** Tabitha Robin, Michael Anthony Hart

**Affiliations:** 1 Department of Applied Biology, Faculty of Land and Food Systems, University of British Columbia, Vancouver, BC V6T 1Z4, Canada; 2 Faculty of Social Work, University of Calgary, Calgary, AB T2N 1N4, Canada; michael.hart@ucalgary.ca

**Keywords:** Indigenous peoples, food systems, wellbeing, knowledge systems

## Abstract

This paper explores the crucial role of Cree elders’ knowledge in revitalizing Indigenous food sovereignty, focusing on food as a cornerstone of Cree identity, spirituality, and wellbeing. Based on a study of Cree elders in Manitoba in 2020, this paper highlights the depth of Cree food knowledge, intertwined with spiritual practices, language, and land ethics. Using an Indigenous research paradigm, ten Cree elders were interviewed and shared their experiences of traditional communal practices, the detrimental impacts of colonialism on food systems, and the spiritual connections between food, land, and community. The elders emphasized the need for education and the preservation of Cree languages, which encode critical knowledge for sustaining food practices. Through their stories, elders illustrated how food sovereignty is not merely about physical sustenance but involves maintaining sacred relationships and responsibilities to the land and all its inhabitants. This research underscores the importance of Cree knowledge in reclaiming and sustaining Indigenous food systems, essential for the health and resilience of Cree communities.

## 1. Introduction

There is a growing movement of Indigenous fishers, growers, seed savers, harvesters, medicine people, activists, knowledge holders, and elders active on the lands of Turtle Island in the practice and pursuit of Indigenous food sovereignty [1,2,3,4,5,6]. Indigenous food sovereignty refers to the activation (via movements, governance, and knowledge systems) of self-determined, ancestral, and land-based food practices by Indigenous bodies through upholding ancestral responsibilities to creation [7,8]. These practices have been found within Indigenous communities since time immemorial and have survived despite a robust colonial infrastructure that has sought to deliberately displace Indigenous bodies from Indigenous lands and to erase Indigenous cultures from Indigenous bodies [9]. While food knowledges, practices, and processes have persevered and adapted, as evidenced by Indigenous peoples’ survival across the globe, colonialism has resulted in significant barriers to access to knowledge systems, practices, and processes [10,11].

While much attention has been paid to the practices of an Indigenous food system (i.e., gardening, hunting), less attention has been paid to the underlying knowledge that supports, shapes, and maintains these practices. For example, Indigenous hunters practice natural law on the land, which means that respectful protocol is taught, modeled, and activated within and across generations [12]. Underlying this knowledge is more knowledge, spiritual practices, and attention to stars, dreams, stories, songs, visions, and interactions with the land, including a deep relationship with plants and animals [12].

Indigenous food skills and knowledge have been deeply affected by a series of overlapping crises following colonization (e.g., endangered languages, climate emergencies); however, this knowledge is necessary and vital to the revitalization of Indigenous food systems. Based on a research project that examined how Cree elders incorporate food into their helping and healing practices in Manitoba, we present examples of how Cree knowledge can provide guidance for how to eat well and be well. This paper pays specific attention to what is described as food knowledge, though it is important to recognize that from an Indigenous worldview, the interconnections and interrelations between all things mean that knowledge cannot be viewed in isolation. Food knowledge is not separate from other knowledge about land, spiritual practice, culture, and governance.

### 1.1. Who Are the Cree?

Cree First Nations are largely found in northern parts of North America (Alberta, Saskatchewan, Manitoba, Ontario, and Quebec). The origins of the term Cree are derived from a French term Kristinon, “a derivative of the Anishinaabe term kiristinō” that described Cree speaking people in northern Quebec in the mid-seventeenth century, although, it is not a term that Cree people use to describe themselves [13] (p. 10). Ininew Elder Dream Keeper Wilfred Buck [12] explains

“Ininew is how my people refer to themselves. Ininew (singular) or Ininewuk (plural) refers to the phrase MIXING OF FOUR. It derives from the root word newo which is the number four. Thus, we are a mixture of four: earth, air, water, fire, or: physical, emotional, mental, and spiritual; or four parts, Plains Cree, Woodland Cree, Swampy Cree and Rocky Cree” (p. 12).

As Cree populations moved westward “their ways of life were influenced by, and in turn influenced the ways of the tribal groups they met. The merging of social groups and economic structures through intermarriage and trade was a logical outcome of the interface between an existing society and a peacefully encroaching one” [14] (p. 150). The Muskéko Ińiniwak, Néhithawak or who some call Asiníkáwithiniwak, and Néhiyawak all reside in parts of what is now known as Manitoba: “These references respectively reflect the “n”, “th”, and “y” dialects of the Cree language” [13] (p. 10). Cree is also one of five linguistic groups in so-called Manitoba. The foundational foods of Cree culture reflect their northern Manitoba geography: whitefish, deer, elk, moose, goose, blueberries, raspberries, and strawberries are common traditional foods found in Cree territories.

Cree people and their communities in Manitoba have experienced significant changes to their food systems due to colonization and resource extractivism. Forced and enforced movement and lack of movement such as the imposed reserve system and the related pass system, which attempted to confine First Nations peoples to the reserves, are common experiences for Cree people. In addition, most Cree communities in Manitoba have been deeply affected by hydro development [15], which has altered the physical, social, cultural, and economic landscapes of these communities. Some communities have been displaced due to the flooding associated with hydro development [16].

### 1.2. Colonialism and Hunger

Prior to colonization, hunger was not a chronic concern for most Indigenous communities [17]. Indeed, Indigenous peoples lived in active and harmonious relation with the land, and in turn, the land provided all that was necessary for survival. Colonization brought with it a collision of jagged worldviews, as Little Bear [18] describes, where Eurocentric views of land as a commodity dominated settler and Indigenous relations. The intense desire and need for colonizers to clear the lands of so-called Canada—of Indigenous peoples and the plants, animals, and water necessary for survival—to support agriculture and settlement brought considerable hardship to Indigenous communities [19,20]. During the 1700s and 1800s, key animal species such as the bison, wolf, and beaver were hunted and poisoned to near extinction, their bodies wasting on the ground, while hunger set in for Indigenous communities [21]. This hunger was weaponized and used as a bargaining tool for treaty negotiations. Indeed, many chiefs signed treaties to alleviate hunger though promised government rations. Sadly, these rations were often withheld to control communities and, in other instances, were rancid [20].

Starting in the 1880s, children were taken from their homes, in many instances forcibly, and sent to residential schools in order to assimilate into Christian culture, where the hunger continued. According to residential school survivors [22], the food provided at the schools was not adequate in quantity or quality, and hunger was pervasive. Mosby and Galloway [23] explain the typical residential school diet was “characterized by insufficient caloric intake, minimal protein and fat, severely limited access to fruits and vegetables, and frequent bouts of food-borne infection” (p. 1044). At the same time, children’s connection to land-based foods and the associated knowledge and skills were nearly severed, as children were removed from their families and communities leaving few, if any, children to receive the teachings of Cree elders and knowledge keepers. This reflected the oppressive commitment of the Canadian government to use the residential school system to sever the tie between children and their families and disrupt the transfer of Indigenous knowledge and practices across generations. A recent study evaluating residential school entrance examination records in Manitoba and Saskatchewan found that Indigenous children entering into the schools from 1919 to 1953, had “substantially lower rates of overweight and obesity (10.9%) than both non-Indigenous (32%) and Indigenous (56.3%) children and youth today” [24] (p. 5). Yet today, the legacy of residential schools can be found in higher rates of diabetes, obesity, and diet-related diseases [23].

### 1.3. Nutrition Experiments

Between 1942 and 1952, a series of nutritional experiments were conducted in Indigenous communities, including the Cree communities of Cross Lake and Norway House. Despite knowledge of widespread hunger in Indigenous communities, test subjects were given supplements instead of food. Historian Ian Mosby [25] shares: “bureaucrats, doctors, and scientists recognized the problems of hunger and malnutrition, yet increasingly came to view Aboriginal bodies as “experimental materials” and residential schools and Aboriginal communities as kinds of “laboratories” that they could use to pursue a number of different political and professional interests” (p. 148). These experiments did not improve health outcomes of Indigenous peoples and were used by government officials as a means to control “the Indian Problem” [25] (p. 148). Similar experiments occurred at six residential schools, where nearly 1000 children were used as test subjects in nutrition experiments between 1948 and 1952 [23].

Though considered early nutrition interventions, these interventions were horrific. Other interventions included regulations of Indigenous peoples and their use of the Family Allowance Act. While non-Indigenous Canadians were free to collect their allowances as cash, Indigenous peoples were given in-kind payments to support the purchase of pre-approved, “high nutritive” foods such as canned tomatoes, pablum, pork meat such as Klik or Spam, dried prunes and apricots, and cheese [25]. Through other church-based assimilative programs, Indigenous women were indoctrinated to learn that food from the land was “improper” and that only food from the kitchen was acceptable [26], leaving behind a legacy that colonial foods such as flour, sugar, and pork were “good”, while moose, muskrat, and berries were “bad”.

Not surprisingly, the legacy of colonial hunger continues today. The disproportionally high rates of food insecurity and the “transition from traditional to industrialized diets has increased the prevalence of obesity, type 2 diabetes and other diet-related chronic diseases globally, but especially for Indigenous populations” [27] (p. 2). This bleak and oppressive history hides important practices that reflect some Cree people’s efforts to maintain a Cree understanding and experiencing of the world and the practices that help the people maintain a sense of hope for their wellbeing and future generations. It is these understandings that this research project attempted to bring forward more openly.

## 2. Materials and Methods

The objective of this study was to understand how Cree elders use food in their helping and healing practices. As Cree people, we witnessed how food was used in ceremonial practice for decades and understood that food was spiritually significant. This qualitative story-gathering research was based in an Indigenous research paradigm that privileged Indigenous knowledge through stories and experiences of Cree elders in Manitoba. An important aspect of this research is the reliance on spiritual knowledge. Indigenous epistemologies are subjectively based processes that depend upon knowledge from elders who have gathered intergenerational teachings and built upon them over time [28]. Elders have accessed ancestral knowledge and have spent lifetimes in active relation with knowledge and ceremonial processes that recognize a spiritual foundation to life.

Dian Million’s [29] concept of felt or affective theory was especially important to the research design. We sought to understand affect through deep relationships with the elders. Historically viewed as subjects of the Crown, Indigenous peoples have been *affected* by a colonial food system. Malnutrition and starvation as the output and end goal of colonialism are felt experiences. On the opposite end are spiritual, reciprocal, and responsible Indigenous relationships to food and land; affect, in these instances, has kept Indigenous peoples alive.

### 2.1. Research Participants

The elders interviewed for this research were chosen purposively, based on decades of experience working and living with(in) Cree communities [30]. Many of the participants were previously known to me through ceremony and food work, which created a strong sense of relational accountability. The interviews for this project took place from February to June 2020. The global pandemic meant that my previous plan to spend time in community with elders had to be re-routed to phone-based interviews. I spoke with a total of ten elders (six male and four female) between the ages of 60 and 80; one in person before the pandemic and nine via phone after lockdowns were imposed. The interviews lasted between one and five hours. Some elders chose to have their names used, while others requested anonymity; they were assigned random pseudonyms. Interviews were recorded on a recording device and transcribed by hand to help situate the lead researcher closer to the data, as per a relational ontology.

### 2.2. Ethical Considerations

Approval for this research was granted by the University of Manitoba Fort Garry Campus Research Ethics Board (#P2019:156). There are other important ethical considerations in presenting this work, however, that go beyond institutional requirements. Indigenous research, or rather, research on Indigenous peoples has historically been a dangerous endeavor for Indigenous peoples. Linda Tuhiwai Smith [Maori] [31] describes how little is actually known about Indigenous cultures: “It galls us that Western researchers and intellectuals can assume to know all that is possible to know of us, on the basis of their brief encounters with some of us” (p. 1). Further damaging are the ways Indigenous knowledge has been subject to distortion, misinterpretation, and romanticization [31,32,33]. Indigenous ethics direct us to affirm that we are properly reflecting on what has been shared with us; hence, each interview transcript was provided to the elders for review and to confirm the content. At the same time, we recognize that what is presented here is deeply influenced by my own subjectivity, in that it is interpretation of the stories that the elders shared with me. They have provided their blessing for my interpretation, but it is my interpretation, nonetheless.

Based on the works of Absolon [34] and my previous work with symbols [6], metaphor was used for analysis. Three metaphors were determined based on the concept of feeding as affect: feeding ourselves, feeding our ancestors, and feeding our communities. The elders reviewed the analysis and deemed the metaphors appropriate. The metaphor of feeding was important in capturing more than the physical aspects of food or being fed. For Cree peoples, we are fed through more than food. We are also fed through our relations and actions; being and doing. The focus of this paper is feeding via food knowledge, ceremonies, and the Cree language and how these examples of Cree knowledge contribute to being well.

## 3. The Findings

The incredible depth and breadth of Cree knowledge that pertain to Cree food systems cannot be adequately captured in this writing. There are maps in the stars that tell stories about Cree relationships to the animals, nutritional guidelines contained within the Cree language, ways of honoring the life of the moose post-hunt, and practices about caring for one another in Cree ceremonies. All of these maps, guidelines, and practices reflect and contribute to Cree knowledge of food systems. Examples of this knowledge and how this knowledge supports and upholds being well for Cree peoples are shared through the stories and experiences of the elders, as follows.

### 3.1. Food Is History: The Context of Hunger

Though discussions with the elders were focused on food in helping and healing capacities, the elders’ spoke at length about the changes they had seen in their food systems, families, cultures, and livelihoods as a result of colonialism. All of the elders spoke about spending time on the land as children and described family and communal settings such as camps where everyone had a job to do. Cooperation and participation are necessary to obtaining food from the land, as Teresa shared: “I grew up at the trapline. Every spring and fall we would all go there and we just ate traditional food so it was always about prepping food”. These communal and ancestral practices formed a way of life, one that was significantly altered by colonialism and settler conquests for land as a resource. Nathan shared how changes to traditional food systems have meant market-based foods are the norm for Cree communities:

“Today much of the hunting, fishing, and gathering areas are no longer as productive as long ago. Settlers have made sure of that. Lands are fenced off; rivers are being dammed up and food harvesting areas have become very difficult. It’s a lot easier to go to store to buy food instead of trying to go find traditional food off the land.”

Many of the Elders spoke specifically of how hydro development damaged and, in some instances, eradicated land-based food practices in Manitoba. Indeed, hydro development (and the associated culture of resource extraction and its infrastructure) has a long legacy for Cree communities in Manitoba. Victor shared the changes experienced by his own family:

“When I was a child, we pretty much lived completely off the land. My grandfather was a self-sufficient person and he provided for his family and his extended family.... he had his family, his brothers, his wife, his children, his brother’s children, their wives, their family. He provided for everybody. And he made a living off the land. And so that’s how we lived, we lived from the food off the land. And when Hydro development started occurring in the 50 s and the 60 s and the dams were put up on the Saskatchewan River, in Saskatchewan and in Manitoba, at Grand Rapids and that flooded out all the people that survived on the land. Right from Saskatchewan to Grand Rapids, people all along that river system were flooded out and they couldn’t provide anymore. So, my grandparents moved everybody into the town of The Pas and we lived on the outskirts of the town of The Pas in a Métis settlement called Umpherville. And he tried to fish and it was really hard fishing and my dad went to work with the CNR [Canadian National Railway]. And he got into alcohol. And shortly after that my mother got into alcohol and pretty much, we got taken away or given away to other people. So, I have 8 siblings and I never grew up with one of them. And two of them I knew about and the other 6, I didn’t know they were there at all. Until one of them had come looking at my door when she was 40 years old. And so, this is one of the changes that happened in regard to living off that land.”

The imposition of the colonial food system, a western food system heralded by colonizers as necessary for correcting the heathen-like aspects of land-based food systems such as hunting, trapping, and harvesting [35], created further damage to Indigenous lives and livelihoods. The lasting effects of these changes, via assimilation, dispossession, and an introduced dependency, are still visible today. Lily offered insight into how interconnected all aspects of Indigenous life are:

“And then they [Indian Agents] started restricting our movements and not being on the trapline as much, people couldn’t go and live on there, different camps, like seasonal camps, because they wanted everybody to stay on the reserve and send the kids to school. And so, they couldn’t follow that lifestyle anymore and when that happened, they started to lose their freedom of movement and in it the will and the ability to live off the land because now they’re stuck on the reserve and started to rely on Hudson Bay, canned and processed foods, and how it impacts not only that ability to be able to support yourself but also to live off the land. Kids were being taken away to residential school, that whole impact not only affected the spirit, the emotional well-being of the community, but the physical health as well. The movement, the exercises, when they were going out trapping, living off the land. So now they have a more sedentary lifestyle.”

Similarly, Jeff described how government regulations altered relations to the land and affected the ability of Indigenous peoples to care for themselves in the ways that they always had.

“And we’ve gone away from those medicines because now we’ve been partially assimilated and it was by law that we could no longer harvest and pick medicines. And I remember my grandfather said, he remembered being in a sermon in the United Church that he was told by the minister, the UC minister, saying before I tell you, before I speak of this sermon today, I received a letter by her Queen Majesty accompanied by the Lieutenant Governor. And this was in August 1935, “you people of Cross Lake will no longer be allowed to harvest or pick any herbal medicines. If you are caught picking medicines, you will be dealt with in the most extreme”. So that tells you a lot what happened, eh. And what we’re trying to attempt to get back. A lot of the ceremonies, a lot of the harvesting actually went underground. And I’m very grateful for my ancestors for taking this underground, keeping it a secret. And now it’s time for us to no longer fear that process, eh? And you hear these sad situations such as that. But evolving through time we have survived with our languages, a way of life and continue to maintain the food sovereignty with what little lands we have left, eh?”

This contextual knowledge contains Cree histories, and according to the elders, the effects of colonialism on Cree wellbeing is part of our present. The elders understand ill-health, diet-related diseases such as diabetes, and poverty in contemporary times to be a continuation of the effects of colonialism.

### 3.2. Food Is Spirit: Ceremony and Spiritual Knowledges

From a Cree perspective, food is not merely physical sustenance or a group of nutrients but rather a physical and spiritual connection to self, ancestors, communities, and land. From ceremonies that celebrate and honor changing seasons or the arrival of the geese in the spring to harvesting medicines on the land and feasting in community, food is sacred. Thus, the activities, practices, and processes of a Cree food system are spiritual endeavors. Andrew explained:

“When we eat food, there’s a physical aspect of how it nurtures the body, but there’s also the spiritual aspect of how that food nurtures your spirit. So, there’s a physical and then there’s a spiritual reflection of that physical, you know. And so, same thing with the sun and with the trees, we understand everything has spirit, right?”

From a Cree perspective, eating food from the land is critical to being well—and to being Cree—because the land contains spirit. Indeed, Ed shared that the land is where the spirit is the strongest:

“Like I said, that’s where the spirit lives. More so, that’s where the spirit is at its strongest.... You can feel it in the rain drops hitting your head, hitting your face. You can feel it in the wind brushing through your hair. You can connect to it from the sun’s rays. The beauty of the night sky. I mean, there’s spirit, there’s life, you can see it.”

Land and all of its inhabitants, including the sun, moon, stars, and wind, is the place where spirit is the strongest. Nora described how Cree understandings of healthy food mean that food comes from the land. Food from the land is free of processing, additives, hormones, and chemicals, but most importantly, it contains good medicine or spirit that is nourishing:

“We’re always mindful that the food that we eat today, that we buy from Safeway or that we buy from Walmart, it’s not good for us. It’s always in the back of our minds. And so that when we have a chance, when we go and travel and have every opportunity to eat fish, to eat moose meat, to eat ducks, to eat moose nose, to eat whatever that’s wild, we do that because we know that it comes from the land. And we know that food that we eat that comes from the land, it was gotten in a good way. You know, the blessings were there.”

Thus, when consuming food from the land, one is nourished both physically and spiritually. Eating food from the land means eating food that was consumed by one’s ancestors and signals a deep and reciprocal connection to the land. The blessings Nora describe exist in the spirit realm and indicate that prayers and offerings were provided for the food. Spiritual nourishment happens through, with, and on the land and with each other. Spiritual acknowledgments and expressions of gratitude for life have been practiced in Cree communities since time immemorial. All of the elders discussed food in ceremonies, including feasting, fasting, sweat lodge ceremony, full moon ceremony, night lodge, smoke lodge, sun dance or thirst dance, naming ceremony, moon time ceremony, strawberry moon ceremony, bear lodge, pow wows, and rites of passage such as walking-out ceremony and berry fasts. Eric shared how ceremonies that bring people together to share food are part of eating and being well. These practices connect people to food, the land, and our ancestors. Connection to spirit is a vital component of a Cree food system.

“Every time we sit together, having a meal together, we are, we are connected…. We acknowledge the food that was prepared and also the people that prepared the food. And we acknowledge that we wouldn’t be alive without the animals that have given up their lives for that food, so we can eat it. And so, we acknowledge that part, eh? So that puts us in communication, in communion with the spiritual laws that remind us that we are connected to the animal world and the plant world. We’re connected to the water and all those things are made, are part of food.”

Spiritual understandings of food recognize that food contains relationships and that food is a relationship [36]. We are surrounded by relationships, and for Cree peoples, relationships to the natural world are critical to health and wellbeing. Relationships and the practice of renewing and maintaining relationships is also critical to one’s ability to access food from the land.

### 3.3. Food Is Treaty: Land Ethics

The elders spoke of food and the land with reverence. They shared that Cree relationships to the land are foundational for the reclamation of Indigenous land-based food systems. Land is a source of spirit, as described above, but Indigenous relationships to the land also reveal an ancestral commitment towards caring for the land and its inhabitants. Nora shared how Cree relationships with the land are reciprocal and begin with an offering:

“So anything that I do, I always, first and foremost I have my tobacco, I use tobacco and I use my prayer cloth and I send out prayers and ask for guidance and direction that the work that I am expected to do, I know that it comes from the Creator.”

In order to take from the land, one must first give to the land, through tobacco, prayer, and ceremony. Offerings are just one example of the deep ethics that Cree peoples practice with the land. Respectful and ethical harvesting protocols and practices also ensure the plants and animals will continue to give themselves to Cree peoples as food, as Andrew shared:

“It’s not only, yeah, it’s necessary but there was a sacredness to it in that these plants and these animals, fish, they made a covenant with Creator to give themselves, to offer themselves to us. So, but there was a, there was like things to that would happen like hunting laws, right, they would talk to you about them. You know, what to hang on the tree after you’re done with the bones or what certain bones can go in the water, which ones don’t. You know, do you point the antlers towards the home? When you’re eating do you pray with your, talk to your relatives before you go take their life, you know?”

The specific ethics of land-based harvest vary from nation to nation and species to species, although there are commonalities. Practices such as taking only what one needs, asking the plant or animal for consent prior to harvest, offerings of tobacco and other sacred medicines, and protocol around ethical harvest (i.e., when to harvest, how to hunt in such a way to avoid wounding an animal) are part of Cree knowledge systems. In the context of medicines, Lily described how these protocols add “value” to the plant medicines themselves:

“Yes, the tonics that we make are all naturally growing plants that we harvest in a very specific and careful way to ensure that they’re at the optimal health value, medicinal value. We’re careful where we harvest medicines and how we harvest them and we ensure that we follow traditional protocols in the harvesting of all the medicines. And yes, those roots, those plants, those berries have very strong traditional value and so we try to ensure that.”

Lily’s statement reveals a deep history of relationality with plants and plant knowledge. That one knows precisely when to harvest and how to harvest, store, and prepare food and medicines is an invaluable skill. These practices help to ensure the future of these foods and medicines and speak to a kind of “sustainability” that has been practiced in Indigenous communities since time immemorial. Colonial interferences have affected the transfer of knowledge around these practices, however. From the pass system and religious indoctrination to residential schools and modern-day child welfare programs, gaps in knowledge transfer—many of which were deliberate tactics from the state to sever connection to the land—have affected Cree communities in Manitoba and indeed Indigenous communities around the world. The elders spoke of the importance of educating youth, in order to maintain these knowledges and practices for future generations. Jeff explained:

“And our understanding from our Creation story, and we do have a Creation story. And that Creation story is that we came from the spirit world and becoming to live a life here in this world, that we have a process to and a responsibility to teach the ones how to connect to the land.”

The emphasis on education was echoed by Dawn, who discussed her work with youth:

“And then I also talk about living off the land because that’s the way I grew up. And that, we should respect our land. And the proper ways of going out hunting for food. And that’s the way we survived when I was young. And to let the kids know that, how would I put it? That everything out there isn’t, you should work towards what you want.”

This education is also cultural and happens relationally, with family, on the land and through upholding responsibilities. Teresa describes her brother’s first moose kill:

“It was a milestone; do you know what I mean? So now they have the skills to kill a moose and I remember when my oldest brother killed his first moose and then my mom was just dancing and she’s making all these calls to my aunts, you have to come. Well, everybody needed to come. They didn’t have to be asked, they knew what was going to happen. And they did prepping. And then when they brought the moose everybody was dancing, being really happy and yeah. Because of the kinship system, my aunts, they treated my brother like royalty. So, it was such a big thing in the community, in the family. Because that’s a role, that’s a responsibility he has, right? Now he can provide for the family and take care of the family.”

These stories reveal the long, sacred, and (re)generative nature of relationships to the land, in which natural law and treaties between humans and all other living things were central to survival. From a Cree perspective, wellbeing is relational and tied to the wellbeing of plants, animals, and their environments. Food is a gift that is the result of those good relationships. Through agreements or treaties that ancestors made with plants and animals, Indigenous peoples were given responsibilities to care for their food.

### 3.4. Food Is Language: You Are What You Eat

All of the elders spoke of the importance of revitalizing and maintaining practices of Cree languages. Cree knowledge must include Cree languages, which are derived from the land. Or rather, the land contains the languages, which contain the knowledge for being well. Jeff described how the land and language are indelibly linked and that food sovereignty begins with *pimatisiwin*:

“But what that particular language tells us, *pimatisiwin* what we are following, so Mother Earth as it uncovers and as it grows. We’re following it, eh. And that was the harvesting part of it. In connection with the constellations and there are many constellations that we know at a certain given time that those strawberries are only here for a period of two, two and a half weeks to three weeks and they’re done. And if you missed the boat, then you’re not following the land. Then you tend to want to eat something else. So, it was a meticulous process for us to stay connected to that ceremony. That’s why they have berry ceremonies and what not. So hence the word, the Creator’s law that we follow as to the connection to the homelands. So, that’s what *pimatisiwin* means, it’s not just a simple word that means life. Well, what is life? It’s such a vague term. And it’s about choices. Ours was not a choice, it was set in motion already. We needed to go harvest this particular berry, we needed to harvest this particular berry and so forth. That’s how we need to understand the connection of food sovereignty and being spiritually healthy, eh.”

The elders explained how many of the spiritual and physical connections of food are not fully understood today due to loss of language practices and associated teachings surrounding relationships to the land and waters that are contained within the language. Jeff explained why Cree language is so integral to Cree food systems:

“Because prior to us coming to this world, our Creation story, that the animals were here first preparing the vegetation, the berries, and so forth, eh? They all had their work cut out. That’s why these animals play a strong part in that role of healing. They’re just not a buffalo; they’re just not a bear. Those kinds of terms are foreign to us. But when we say them in the language they become so connected to an individual and they’re able to understand.”

(Jeff)

Andrew further described how the languages provide direction for eating well:

“The language coming from the land, the spirit of the land, the spirit of the water, the spirit of the sky, and therefore that grew with our understanding of our spiritual relationship with those physical, natural relatives. And so, when you start to understand the original stories of them and the evolution of the language then you start to understand what you’re feeding yourself with more.”

Jeff explained how critical this understanding is: “Berries were always a part of our natural substance in maintaining a healthy balance within our First Nation people, however, how they are translated is what is in question”. Take, for example,

“*ininimina*, those are blueberries. But what it really means is *inninew minum*, what it translates to is the health of our people lies in those berries. *Inninew* is a human being, *minomiyamum* is health, the good life. *Ininimina* is those two words together to make the word blueberry. Now knowing that ininimina that it translates to the concept that the health lies within these berries, our life, our well-being lies within these berries. It’s significant. Contrast that with blueberry. What does the word blueberry tell you? It doesn’t tell you anything.”

(Ed)

Similarly, Jeff explained the strawberry:

“So, when we talk about the strawberry, we call it *mitemina*. *Mitemina*. The very first implication to that prescribed language, what it’s telling us, is to have a healthy heart. So, you don’t have any, you know, you don’t have any blood clots. And it’s not these strawberries that you buy at the store that they inject and grow like apples, it’s the tiny ones that come from the Earth, eh. Then we go on, all these, all these berries have a purpose to them.”

In Cree, all of the berries are named after how they are used by the body and spirit. While evocative, the elders describe how we must include the language in the recovery of our food systems, not just the direct translation to English, but rather the most fulsome understanding and translation. The berries contain instructions for how to eat to be well.

## 4. Discussion

### 4.1. Food Is More than Sustenance

Food is often presented in the literature as a means to avoid food insecurity and a conduit to receive nutrients [37]. While food insecurity is undoubtedly critical to being well, in an Indigenous context, food is much more than sustenance. This has particular resonance for Indigenous peoples, whose land-based and ancestral food systems have been under attack since colonization. Indeed, the eradication of land-based foods and the introduction and enforcement of a colonial food system speak to the power inherent in food: as a tool used to subjugate and assimilate Indigenous peoples into western culture [35]. The dark and disturbing details of the history of food for Indigenous peoples, see ([19,20,25]), and most certainly Cree peoples across Manitoba is in stark contrast to the ways that the elders understand and incorporate food in their helping and healing practices.

Food, as the elders shared, contains spirit. It is medicine, it is a source of connection and reciprocity to the land and our ancestors, and it is the means of passing cultural knowledge generationally. Food is economy, livelihood, and the means of caring for oneself, families, communities, and nations. Other Indigenous authors have echoed these notions: food as a source of wellbeing [38]; food as place-based ecological knowledge [39]; food as currency, through the revitalization of historic trade processes [40]; food as sustainable self-determination [Cherokee] [41]; food as connection to traditional territory; and food as “relationship to plants and animals that give themselves as food” [42] (p. 12). Food systems engagement (i.e., hunting, filleting, and harvesting medicines) can also support strong connections to Indigenous identity and senses of belonging [6,43] and are deeply physical activities “conducive to the maintenance of cardiovascular health” [44] (p. 119).

For Cree people in Manitoba, like many Indigenous peoples, food is found in all aspects of daily life. It is contained within Cree cosmologies, ontologies, epistemologies, languages, and spiritualities. What is apparent from this research is that food, from a Cree perspective, is connected and interconnected to all elements of Indigenous life. For Indigenous peoples, food systems (i.e., the land, air, water, sky, sun, moon, etc.) and the practices and processes of food systems are the foundation of being well. Food is used in a multitude of Cree ceremonies, as stated by the elders; feasting, fasting, sweat lodge, full moon ceremonies, night lodge, smoke lodge, sun dance or thirst dance, rites of passage such as walking out ceremonies and berry fasts, naming ceremonies, moon time ceremonies, strawberry moon ceremonies, bear lodge, and pow wows all incorporate food. Food is not merely sustenance for Cree peoples, but rather a practice, a way of life, that signals reverence for and commitment to these gifts from creation. This way of life is Indigenous food sovereignty.

### 4.2. Cree Knowledge as Food Literacy

The food concepts that the elders shared describe the practices and processes of eating well and eating for wellbeing. Some of these concepts were contained within the Cree language. Importantly, as the elders explained, their meanings extend beyond direct translation: as Ed stated, ininimina are not just berries that are blue. In learning the translations and meanings of the berries, it is clear that the language contains guidance for how to eat. Nutrition literacy refers to an “area of competence upon which healthy dietary behaviour depends” [45] (p. 379), while food literacy is described as including “the knowledge, skills, and practices that enable citizens to participate more effectively in a sustainable and equitable food system” [46] (p. 195). Both are closely tied to the concept of health literacy, which is sometimes used interchangeably with food literacy (for a more rigorous debate on terminology, see [45]). Regardless of the terminology used, nutrition education is a blooming field, and Indigenous peoples, especially those with diabetes, are often the targets of such undertakings [47].

The idea that Indigenous peoples require food literacy, nutrition literacy, and/or health literacy can be traced back to colonization and ongoing colonialism [35]. When the government destroyed traditional food systems and legislated poverty, rations were introduced to Indigenous communities. Notably, these rations were foods that were foreign to Indigenous bodies. Flour, cheese, milk, pork, and pig lard were introduced foods, as were sugar and salt. Patterns of shame around eating began early, as Indigenous peoples were told their diets including breast milk were inferior, and they, as a people, were in need of civilization [20]. Mosby [47] shares the government’s stance on northern Cree peoples’ malnutrition and starvation in the 1940s:

“They have abandoned the native eating habits of their forefathers and adopted a semi-civilized, semi native diet which lacks essential food values, brings them to malnutrition and leaves them prey to tuberculosis and other disease. The white man, who unintentionally (The term unintentional here is contentious and at odds with the works of scholars who have documented otherwise (for example, [20,21,25,47,48,49])) is responsible for the Indians’ changed eating habits, now is trying to salvage the red man by directing him towards proper food channels” [47] (p. 50).

Upon visiting these communities and witnessing the malnutrition and hunger, the government and its allies were quick to chastise Indigenous peoples: “shiftlessness, indolence, improvidence and inertia, so long regarded as inherent or hereditary traits in the Indian race may, at the root, be really the manifestations of malnutrition” [25] (p. 147).

The idea of proper eating had little regard for land-based foods and the robust system of spiritual, ethical, and physical knowledge surrounding the foods, further altering Indigenous peoples’ relationships to their food systems. The assumption that Indigenous peoples were, or are, without “essential food values” is dangerous and incorrect. Indeed, throughout this research project, I was awed at the ways that the elders described how food is sacred. It is understood that food deserves and teaches respect, a central value to Cree culture. Indeed, there is a system of knowledge that supports and guides Cree food systems, as described throughout this paper.

The elders explained what Cree diets should consist of: food from the land, food that their ancestors will recognize, and food that is Indigenous to their nation. I think of this in comparison to Michael Pollan’s [50] famous food rules: real food is what your grandparents recognize. I also think of how blueberries are marketed as a “superfood” and part of many heralded healthy diets. Rather than use outside sources and teachers and rather than more interventions by government, academics, and non-profits, the enactment of Indigenous food systems must come from within the community. Community members are key teachers in this way; as language speakers, hunters, trappers, medicine people, and ceremonialists, Indigenous peoples have much to offer in the ways of eating.

## 5. Conclusions

Indigenous food knowledge is values-based and has a theoretical underpinning that includes deep reciprocal connection to ancestral and natural worlds. Elders know that food is a gift from Creator, and they utilize these gifts extensively in their care practices. As part of the gift, Indigenous peoples are to activate, practice, and enact the values of their food and cultural systems. This ensures the maintenance of ancestral responsibilities, responsibilities that are contained within Cree teachings, values, worldviews, languages, and naming systems. These are central components in Indigenous food systems that are often forgotten, yet are critical for being well. That is, to eat well means to be in good relation to one’s food system.

Cree elders know how to be in good relation to their food systems. They understand the significance of food in Cree histories. They also understand the potential of food, via food sovereignty, for Cree futures. Cree knowledge is needed to support food sovereignty. What is apparent from this research is that the pathway should be grounded in and illuminated by this knowledge.

## Data Availability

The data are not publicly available in accordance with OCAP^®^.
